# Supplementary material for: Complexome profiling of the Chlamydomonas psb28 mutant reveals TEF5 as an early PSII assembly factor
Source: Plant Cell. 2025 Mar 17;37(6):koaf055. doi: 10.1093/plcell/koaf055 (PMC12164590; doi:10.1093/plcell/koaf055)
Supplement: koaf055_Supplementary_Data [file koaf055_supplementary_data.zip › psb28_interactive.zip!/plots/intensity/qualityPlots/peptides/_Cre43.g760497.t1.1.html]

### Cre43.g760497.t1.1

Trivial name:   
SeqCoverage [median]: 11.40  
Mass: 28.95 kDa  
  
 

| Sequence | MissedCleavage | Mass [Da] | wtIntensity | muIntensity | Score |
| --- | --- | --- | --- | --- | --- |
| EGEGVAAR | 0 | 787.38 | 368.02 | 1560.58 | 77.28 |
| VLEMSLEEAR | 0 | 1175.59 | 1189.81 | 3758.54 | 166.09 |
| VLETLGADPAK | 0 | 1112.61 | 1845.06 | 5370.47 | 132.01 |
| VLETLGADPAKIR | 1 | 1381.79 | 0.00 | 442.42 | 35.20 |
|  |  |  | 3402.9 | 11132.0 |  |
